# Estrogen hormone is an essential sex factor inhibiting inflammation and immune response in COVID-19

**DOI:** 10.1038/s41598-022-13585-4

**Published:** 2022-06-08

**Authors:** Fuhai Li, Adrianus C. M. Boon, Andrew P. Michelson, Randi E. Foraker, Ming Zhan, Philip R. O. Payne

**Affiliations:** 1 Institute for Informatics (I2), Washington University in St. Louis School of Medicine, St. Louis, MO USA; 2 Department of Pediatrics, Washington University in St. Louis School of Medicine, St. Louis, MO USA; 3 Department of Medicine, Washington University in St. Louis School of Medicine, St. Louis, MO USA; 4 Pathology & Immunology, Washington University in St. Louis School of Medicine, St. Louis, MO USA; 5 Department of Molecular Microbiology, Washington University in St. Louis School of Medicine, St. Louis, MO USA; 6 Pulmonary and Critical Care Medicine, Washington University in St. Louis School of Medicine, St. Louis, MO USA; 7 National Institute of Mental Health (NIMH), NIH, Bethesda, MD USA

**Keywords:** Infectious diseases, Systems biology

## Abstract

Although vaccines have been evaluated and approved for SARS-CoV-2 infection prevention, there remains a lack of effective treatments to reduce the mortality of COVID-19 patients already infected with SARS-CoV-2. The global data on COVID-19 showed that men have a higher mortality rate than women. We further observed that the proportion of mortality of females increases starting from around the age of 55 significantly. Thus, sex is an essential factor associated with COVID-19 mortality, and sex related genetic factors could be interesting mechanisms and targets for COVID-19 treatment. However, the associated sex factors and signaling pathways remain unclear. Here, we propose to uncover the potential sex associated factors using systematic and integrative network analysis. The unique results indicated that estrogens, e.g., estrone and estriol, (1) interacting with ESR1/2 receptors, (2) can inhibit SARS-CoV-2 caused inflammation and immune response signaling in host cells; and (3) estrogens are associated with the distinct fatality rates between male and female COVID-19 patients. Specifically, a high level of estradiol protects young female COVID-19 patients, and estrogens drop to an extremely low level in females after about 55 years of age causing the increased fatality rate of women. In conclusion, estrogen, interacting with ESR1/2 receptors, is an essential sex factor that protects COVID-19 patients from death by inhibiting inflammation and immune response caused by SARS-CoV-2 infection. Moreover, medications boosting the down-stream signaling of ESR1/ESR2, or inhibiting the inflammation and immune-associated targets on the signaling network can be potentially effective or synergistic combined with other existing drugs for COVID-19 treatment.

## Introduction

Globally, by May 20, 2022, over 520 million^1^ people were diagnosed with Coronavirus Disease 2019 (COVID-19), which is caused by the Severe Acute Respiratory Syndrome Coronavirus 2 (SARS-CoV-2). COVID-19 has a relatively high mortality rate^2^, and more than 6.2 million patients have died from the pandemic to-date^1^. The number of patients infected with SARS-CoV-2 and succumbing to COVID-19 and related complications are still increasing rapidly worldwide. Meanwhile, several vaccines have been approved for infection prevention, but may not prevent viral transmission to susceptible individuals. Further, remdesivir is the first FDA approved drug for COVID-19 treatment and it has shown variable efficacy^3,4^. From the perspective of viral replication at the molecular level, remdesivir is the most widely-used drug that specifically targets and inhibits the RNA-dependent RNA polymerase (RdRp)^5,6^, which is the critical target for viral replication^5^. On May 1st, 2020, Remdesivir was granted an FDA Emergency Use Authorization (EUA) for COVID-19 treatment, and obtained full FDA approval on October 22, 2020. The final report from the most recent clinical trial of Remdesivir indicated that is can reduce the recovery time of COVID-19 patients to about 5 days, and that it reduces overall mortality rates. Though these results are promising, the effect of Remdesivir alone remains limited. In light of these challenges in terms of the prevention and treatment of COVID-19, many drugs and drug combinations are being tested on their own and in combination with Remdesivir in more than a thousand clinical trials globally. Among these drugs is dexamethasone, an FDA-approved drug, which was reported to be able to reduce the death rate of patients with severe COVID-19^7^. Recently, the new drug nirmatrelvir^8^, targeting the main protease (Mpro)^9^ target that is also important for viral replication and transcription^9,10^, was authorized by FDA by December 2021 for the treatment of mild-to-moderate COVID-19 patients. However, the death rate for such patients remains high despite such potential reductions.

Sex had been indicated as an essential factor in the mortality of COVID-19. The global data of COVID-19 showed that men have a higher mortality rate than women^11,12^. For example, the meta-analysis was conducted, and the analysis results showed that there were thre times more male patients requiring intensive treatment unit admission than females, whereas there is a similar proportion of men and women with COVID-19 confirmation^5^. In addition, we further observed that the proportion of mortality of female increases starting from around the age of 55 significantly (see the “Results”). Thus, the sex difference indicated that sex related genetic factors could be an interesting mechanism and targets for COVID-19 treatment, combined with existing treatment options. Numerous studies have been reported in the literature concerning efforts to understand the signaling mechanism and identify effective targets for COVID-19 from different perspectives^13–22^, like transcriptomic data analysis, proteomics data analysis, investigation of relevant protein structures, and the use of experimental and computational methodologies. However, few studies have been specifically designed for investigating sex related signaling pathways and targets. The underlying targets and signaling pathways associated with the sex difference remain unclear.

In response to the preceding gaps in knowledge, in this study, the objective is to uncover the potential sex associated factors using systematic and integrative network analysis. Specifically, we explore how protein–protein interaction screening data, transcriptomic data, signaling pathway data, protein–protein interaction signaling networks, and transcription factor-target regulatory networks can be integrated to enable such network analyses. We then go on to determine drug-target interactions and associations based upon an interrogate of the drugBank^23^ and connectivity map (CMAP) databases^24,25^ in order to identify the potentially-effective targets from the drug-target interaction perspective. Our unique results with novel discoveries, i.e., estrogens, like estrone and estriol, (1) interacting with ESR1/2 (estrogen receptors), (2) can potentially inhibit SARS-CoV-2 caused inflammation and immune response signaling in host cells; and (3) the loss of estrogens to an extremely low level after 55 years of age is associated with the mortality increasing in female. These three novel observations support that estrogen is an essential sex factor causing the distinct fatality patterns between male and female COVID-19 patients.

## Materials and methods

### Methodology overview

**Figure 1:**
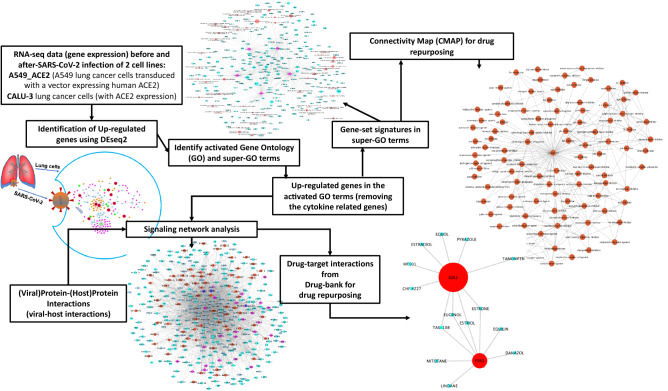
Overview of the proposed methodology.

Figure 1 shows the overview of the proposed integrative data analysis model. Specifically, the raw counts of RNA-seq data (gene expression) of two lung cancer cell lines expressing ACE2 before and after SARS-CoV-2 infection were collected and used to identify commonly up-regulated genes using the DEseq2 model. Then gene ontology (GO) term enrichment analysis and super-GO term (consisting of a set of GO terms with similar functions) analysis were conducted to identify activated biological functions caused by SARS-CoV-2 infection using such up-regulated genes. Subsequently, up-regulated genes associated with super-GO terms were used as the gene set signatures in order to repurpose drugs using the connectivity map (CMAP) database, thus producing a list of top-ranked drugs that can potentially inhibit these up-regulated genes in the activated super GO terms. In parallel, the activated core signaling network caused by viral infection within host cells was constructed by integrating viral-host protein–protein interactions, the up-regulated genes associated with the activated GO terms, as well as the bioGrid protein–protein interaction network, and fold change values of all the genes as found in the RNA-seq data. Then, drug-target interaction information was derived from drugBank, and drugs that target the inferred signaling network proteins were identified as potential candidates for repurposing for SARS-CoV-2 replication inhibition and resultant COVID-19 treatment. The details of the related datasets and methods are described further in the following sections. All database and datasets used in this study are open and publicly available.

### Identification of RdRp protein complex and co-factors of SARS-CoV-2

The 3D cryo-electron microscopy (cryo-EM) structure of the protein complex associated with RNA-dependent RNA polymerase (RdRp), i.e., nsp12, and its two co-factors, nsp7 and nsp8, of SARS-CoV-2, has been reported upon in the literature, including in the indicated references^26,27^. These structures are essential contributors of viral replication, working together with the transcription machinery of host cells. The RdRp is thought to be the primary target of the antiviral drugs, like Remdesivir.

### Identification of proteins binding to nsp7, nsp8 and nsp12 (RdRp)

In the previous study^14^, protein–protein binding assays were conducted to identify the (prey) proteins binding to (bait) nsp7, nsp8 and nsp12 (and other viral proteins). In brief^14^, 26 of the 29 SARS-CoV-2 proteins were cloned, tagged and expressed in human HEK-293T/17 cells; and then the affinity-purification mass spectrometry approach was used to identify the proteins binding to these 26 proteins respectively. Specifically, there are 20, 34, and 24 proteins interacting with nsp12, nsp7 and nsp8 respectively, and there is no overlap among these proteins. More details and the data are available in references^13,14^.

### Transcriptomic (gene expression) data analysis of two lung host cell lines, A549_ACE2 and CALU-3 cells, after SARS-CoV-2 infection

The raw counts of RNA-seq data (transcriptomic response) of A549_ACE2 (engineered with expression of ACE2 protein), and CALU-3 lung cancer cells (with ACE2 expression) cells infected by SARS-CoV-2 were available in a single dataset (GSE147507^19^) from the Gene Expression Omnibus (GEO) database^19,20^ The DEseq2^28^ approach was used to calculate the fold change and p-value of individual genes in the A549_ACE2, and CALU-3 lung cancer cells respectively before- and after-exposure to SARS-CoV-2 (with 3 replications). Then up-regulated genes were obtained by using fold change ≥ 2.0 and p-value ≤ 0.05. Subsequently, the overlapping (intersection) genes between the up-regulated genes found in CALU-3 and A549_ACE2 cell lines were selected for further analysis as described below.

### Gene ontology (GO) enrichment analysis and super GO clustering analysis

To conduct the GO enrichment analysis, the ‘GO.db’ (to obtain the GO term id and names using the ‘Term’ and ‘Ontology’ functions) and ‘org.Hs.eg.db’ (to extract the genes in each GO using the ‘get’ function) r packages/database were used. Then the p-value of each GO was calculated using the Fisher’s exact test, which compares the numbers of up-regulated genes in a given GO and the number of up-regulated genes in the rest genes that do not belong to the given GO. Then the p-values are used to identify activated biological processes (BP) gene ontology (GO)^29^ terms. Specifically, the activated GO terms with genes in [10, 500] and p-value ≤ 0.05 were identified. Among the hundreds of GO terms identified, we further empirically selected the potentially virus-related GO terms. To further cluster the selected GO terms into sub-groups, named super GO terms, which share the similar biological processes, the GO-GO similarity among the selected GOs was then calculated using a semantic similarity metric^30^ (implemented using the GOSemSim R package), and then the affinity propagation clustering^31^ (APclustering) approach was employed to identify the GO sub-groups, i.e., super-GOs. The number of sub-groups was set 5 empirically.

### Drug repositioning using connectivity map (CMAP) and the gene signatures associated with individual super GO terms

The up-regulated genes associated with individual activated GO terms included in each super-GO terms were used as the gene set signatures to identify potentially effective drugs that can inhibit the activated biological function using the connectivity map (CMAP)^24,25^ database. For some super GO terms, there were more than 150 associated up-regulated genes. Since the CMAP suggested the size of gene set signatures should be no more than 150 genes, the genes associated with individual super GO terms were ranked based on the average fold change (of the A549_ACE2 and CALU-3 cells versus the mock condition) in the decreasing manner. Then the top-ranked 150 genes were selected as the gene set signatures to repositioning drugs that can potentially inhibit these up-regulated genes in the gene set signatures. Specifically, the gene set enrichment analysis (GSEA)^32^ was applied on the gene set signatures and the z-profiles (gene expression variation before and after treatment with 2513 drugs and investigational agents in the CMAP database) of nine cancer cell lines to identify gene set signature-specific inhibitory drugs. Based on the average GSEA scores, the top ranked drugs were selected.

### Up-regulated transcription factors (TFs)

Transcription factors (TFs) are important when exploring regulatory networks. To investigate which set of TFs were up-regulated after SARS-CoV-2 infection in the 2 lung tumor cells use in our study, TF information was derived from the molecular signature database (MSigDB)^33^. Specifically, the C3 category molecular signatures were obtained, which corresponded to regulatory gene sets. From those, 415 TFs interacting with 14,802 target genes were identified.

### RdRp-host interaction signaling network inference model

The protein–protein interaction (PPI) network for these genes was derived from BioGRID^34^ and used as a background signaling network. There were approximately 21,699 genes and 368,918 interactions identified using this approach. The RdRp consequent signaling network inference was defined as a sub-network inference problem within BioGRID. Here, we proposed a novel consequent signaling network inference model. Specifically, let ${G}_{0}^{i}=\langle {R}_{i}, \varnothing \rangle$ denote the initialized consequent signaling network of root nodes ${R}_{i}$. The root nodes are the proteins interacting with the nsp12, nsp7 and nsp8. The growth update of the consequent signaling was defined as: ${G}_{t+1}^{i}=f\left({G}_{t}^{i}, {G}_{B},{V}_{K}, d\right)$, where ${G}_{t}^{i}$ and ${G}_{B}$ is the current down-stream and background (BioGRID) signaling networks, respectively. The edge, ${e}_{ij}$ (protein interactions between ${g}_{i}$ and ${g}_{j}$) of background signaling network, ${G}_{B}$, is weighted as: $w\left({e}_{ij}\right)=\frac{1}{abs\left(fc({g}_{i}\right)}+\frac{1}{abs\left(fc({g}_{j}\right)}$. ${V}_{K}$ and is a vector including K candidate target genes (e.g., the activated genes). For any gene, ${g}_{k}\in {node(G}_{t}^{i})$, the shortest paths from ${g}_{k}$ to the left candidate target genes in ${V}_{k1}$, is then calculated. Subsequently, an activation score for each path, *p*_*k,j*_, is defined as: ${s}_{kj}={\sum }_{{g}_{m}\in {p}_{ij}}fc({g}_{m})/n$, where *fc(.)* is the fold change calculator and n is the number of genes on the signaling path. If $n>d$ (search depth parameter) the signaling paths are discarded. In this manner, the parameter k2 decides the search depth. Finally, the signaling path with highest activation score is added to the consequent signaling network. This process is then conducted iteratively until all target nodes are included into the consequent signaling network.

### Identification of FDA approved drugs inhibiting genes on the uncovered signaling network

The FDA approved drug and their target information was derived from drugbank^23^ database. Specifically, the ‘Target Drug-UniProt Links’ file of ‘Approved’ drugs (the FDA drugs) was downloaded from the ‘EXTERNAL LINKS’ tab on the ‘Downloads’ page of DrugBank website to identify the FDA drugs; and associated drug-target information. The genes, with degree ≥ 10, in the RdRp-host signaling network were selected. Then the FDA approved drugs inhibiting the selected genes were identified as drug candidates that can potentially perturb the uncovered signaling network.

## Results

### Activated biological processes in host cells caused by SARS-CoV-2 infection

As described in the “Materials and methods” section, 656 overlapping (intersection) genes were identified between 1335 and 2260 up-regulated genes identified in the CALU-3 and A549_ACE2 cell lines respectively with the fold change ≥ 2.0 and p-value ≤ 0.05. Via GO enrichment analysis, 474 GO terms in the BP category were identified with a p-value of ≤ 0.05, and the number of genes in these GO terms was within a range of 10 to 500. Among these identified GO terms, the well-known inflammation and innate immune related GOs terms were identified. As we described in the “Materials and methods” section, given that we aimed to identify the essential host transcription factors that generate proteins which interact with the RdRp of SARS-CoV-2 and its co-factors for viral replications, these inflammation and immune response related GO terms were filtered out empirically. Subsequently, 96 GO terms were finally selected (see supplementary Table 1), in which the 299 activated genes (out of the 656 genes) were included (see supplementary Table 2). As seen, a large set of inflammation and immune response related signaling processes were activated, which indicated the strong inflammation and immune response. In addition, a set of core signaling pathways, like PI3K, ERK1/2, NFkB, JAK-STAT, p38MAPK, JNK, were activated, which are the potential therapeutic targets, like JAK2, for identifying potentially effective COVID-19 treatments. Among the 299 genes, 25 were transcription factors, including: ATF3, ATF4, EGR1, ETS1, FOXO1, FOXO3, GATA3, GATA6, IRF1, IRF2, IRF7, MIER1, NFKBIA, NR1D1, NR4A2, PER1, RORA, RREB1, SKIL, SMAD3, SNAI1, SOX9, STAT1, STAT5A, ZNF175, which indicated the strong effects of SARS-CoV-2 on host regulatory signaling.

**Figure 2:**
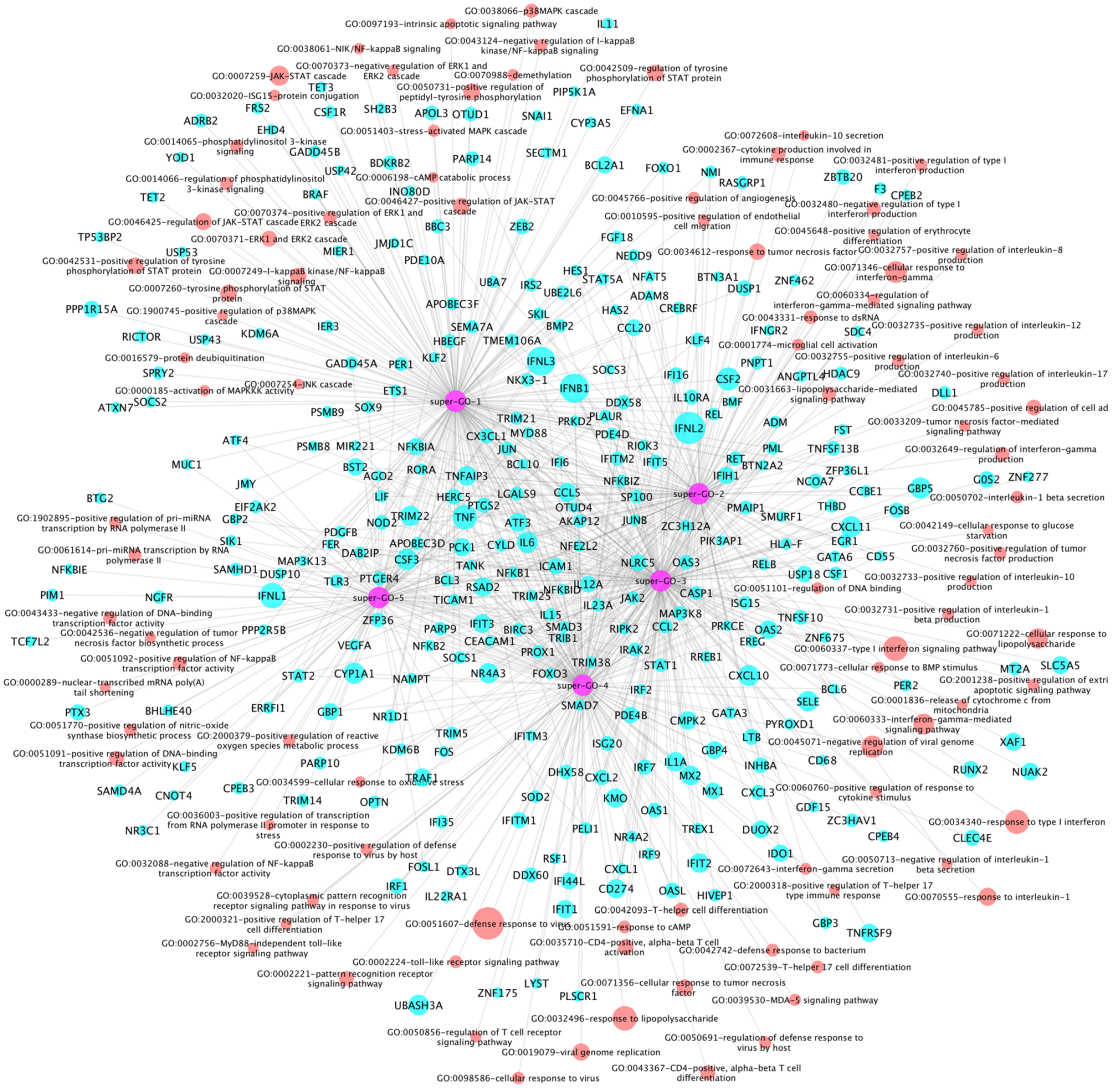
Network of Super GO terms, GO terms, and the associated up-regulated genes, which includes 5 super-GOs (purple), 96 activated GO terms (red) and activated 299 genes (cyan).

Further, the 96 activated GOs were clustered into 5 sub-groups, i.e., super-GO terms (see Fig. 2). There were 25, 20, 21, 19 and 11 GO terms in each of give super GO terms respectively. Accordingly, there were 174, 115, 179, 142 and 86 genes associated with each super GO terms respectively. As seen in Fig. 2, in addition to a set of genes uniquely associated with the 5 super GO terms, some genes were associated with multiple super GO terms. More interestingly, in super GO term-1, there were mainly the core signaling pathways, like JAK-STAT, ERK1/2, NFkB, p38MAPK, PI3K. The interleukin and interferon signaling pathways were included in super GO terms 2 and 3. The T-helper, MyD88, and other immune response signaling pathways were presented in super GO term 4. The DNA-RNA binding, biosynthesis, metabolic signaling pathways were identified in super GO term 5.

### Estrogens inhibiting inflammation and immune response in host cells

**Figure 3:**
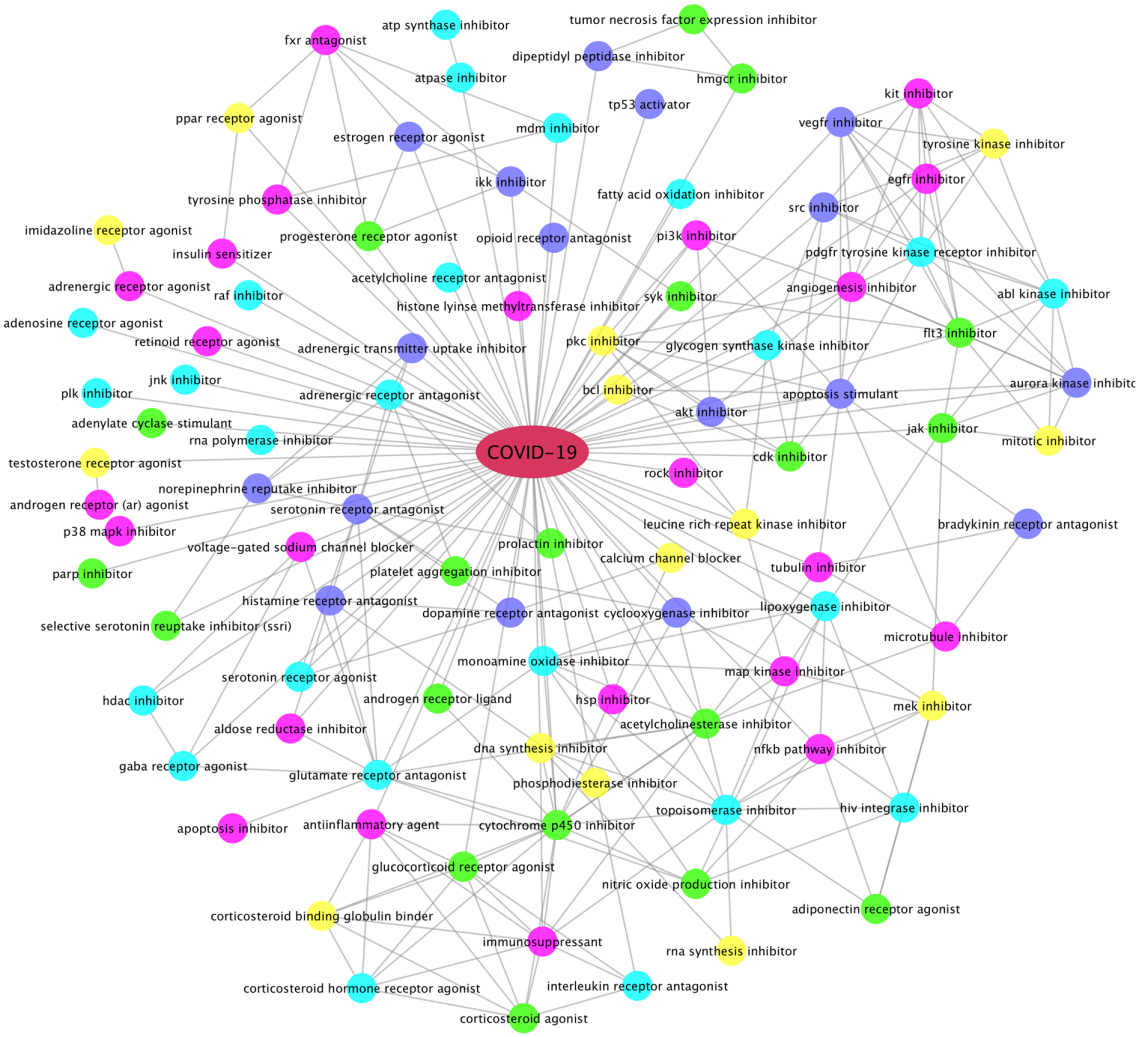
Top-ranked drug categories that can potentially inhibit activated GO terms after SARS-CoV-2 infection. The connection (edge) between two different categories indicates that some drugs can inhibit both categories.

Using the up-regulated genes found in the 5 super-GOs, we identified drugs that can potentially inhibit these activated super-GO terms. Drugs and compounds that had a CMAP drug ranking score of ≤ − 85 (indicating that these drug compounds could inhibit the activated gene set signatures in each super-GO term) were selected. Figure 3 shows the top-ranked drug categories. Interestingly, the estrogens, estrone (E1) and estriol (E3, weak estrogens), which interact with ESR1/2, as well as estradiol, were top-ranked to be able to inhibit the inflammation and immune response-related biomarker genes in the super-GO terms. As seen, there are a diverse set of drug categories, in addition to *estrogens*, calcium channel blockers, gamma-aminobutyric acid (gaba), androgen, serotonin, adrenergic, glucocorticoid receptor agonist/antagonist; inhibitors of JAK2, MDM2, HDAC, SRC, PI3K, EGFR, RAF, JNK, PLK, p38Mapk, VEGFR, MEK, FLT3, CDK, AKT, HSP, NFkB, IKK; HIV integrase inhibitor, DNA/RNA synthesis inhibitors; and tyrosine, map, aurora kinase inhibitors; corticosteroid agonist, immunosuppressants; SSRI (anti-depression) inhibitors, platelet aggregation inhibitors, as well as tubulin, microtubule inhibitors. Some of these categories have been reported in the new studies about potential COVID-19 treatments. For example, gamma-aminobutyric acid (gaba) administration has been associated with severe illness and death in coronavirus infection in mice^35^. Similarly, calcium channel blockers inhibit SARS-CoV-2 infectivity in lung cells^36^. Moreover, the recent clinical trial results indicated that the baricitinib (the JAK2 inhibitor) plus Remdesivir in combination can significantly reduce the recovery time for and mortality rate of COVID-19 patients receiving high-flow oxygen^37^. These top-ranked drug categories can guide the selection of effective drugs or drug combinations for COVID-19 treatment.

Among the top-ranked drugs based on the super GO term gene set signatures, there were 158 FDA approved drugs. And, 13 of 158 FDA drugs had been evaluated in recent clinical trials: azithromycin, chloroquine, dexamethasone, fluvoxamine, formoterol, lenalidomide, losartan, progesterone, ramipril, sildenafil, simvastatin, sitagliptin, tacrolimus, thalidomide and verapamil. Simvastatin^38^, losartan^39^, and chloroquine + azithromycin (used to treat bacterial infections)^40^ have also showed potential efficacy in COVID-19 treatments. SSRI and anti-depression drugs, like fluvoxamine, were potentially effective for COVID-19 treatment^41^. Ramipril is the ACE inhibitor. The drug dexamethasone, belonging to the glucocorticoid receptor, corticosteroid agonist, and immunosuppressant categories, was the first drug reported to be able to significantly improve the mortality rate of COVID-19 patients in clinical trials^7^. Of note, the National Institutes of Health (NIH) have recently initiated drug combination clinical trials including Remdesivir plus dexamethasone or baricitinib (JAK2 inhibitor) for COVID-19 treatment.

### ESR1 and ESR2 were identified in the viral-host interaction signaling network

**Figure 4:**
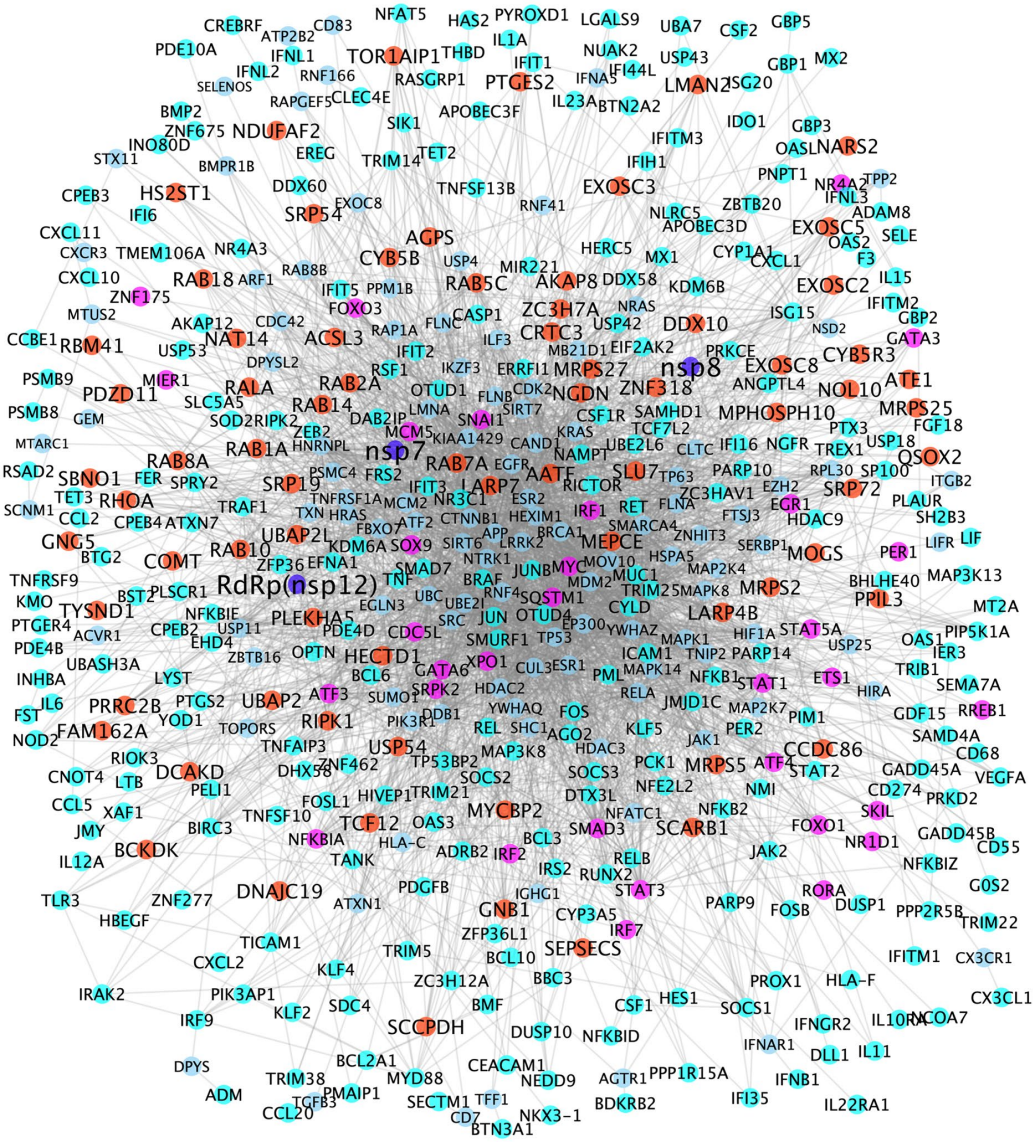
RdRp (nsp12, nsp7 and nsp8)-host interaction signaling network (with 3373 interactions among 486 proteins). Blue, red, purple, cyan and light blue nodes represent the RdRp-nsp7-nsp8, prey proteins (interacting with RdRp protein complex, transcription factors, up-regulated genes, and proteins linking the prey proteins and up-regulated genes.

Two RdRp-host interaction signaling networks were generated using the proposed network analysis model described in the “Materials and methods” section. Supplementary Fig. 1 shows the Nsp12(RdRp)-host interaction signaling network linking the 299 selected genes from 20 prey proteins (root genes) that interact with nsp12 (the RdRp bait protein). There were 22,666 interactions among these 405 genes. Figure 4 shows the Nsp12-nsp7-nsp8(RdRp)-host interaction signaling network linking the 299 selected genes from 73 prey proteins (root genes) that interact with at least one of nsp12, nsp7, nsp8 (the RdRp bait protein). There were 3373 interactions among these 486 genes. There are 395 overlapping genes between the two signaling networks, which indicated that the nsp12-host signaling network is a subnetwork of the nsp12-nsp7-nsp8-host signaling network. As seen in the center area of the signaling networks, ESR1 and ESR2, the signaling receptors of estrogens, were identified in the viral-host interaction signaling network, which indicated that ESR1 and ESR2 play important roles in viral-host interactions. In addition, many other signaling targets, e.g., ESR1, ESR2, TP53, HIF1, MYC, HDAC3, TNF, MAPK, RELA, APP, JUN, MDM2, JAK, STAT, CTNNB1, were identified that intensively linked to other signaling targets. These signaling targets linking the prey proteins can be potentially essential viral-host interaction signaling targets, which may work together to facilitate viral replication and regulate host cell signaling response.

### Drugs directly perturb the viral-host interaction signaling networks

**Figure 5:**
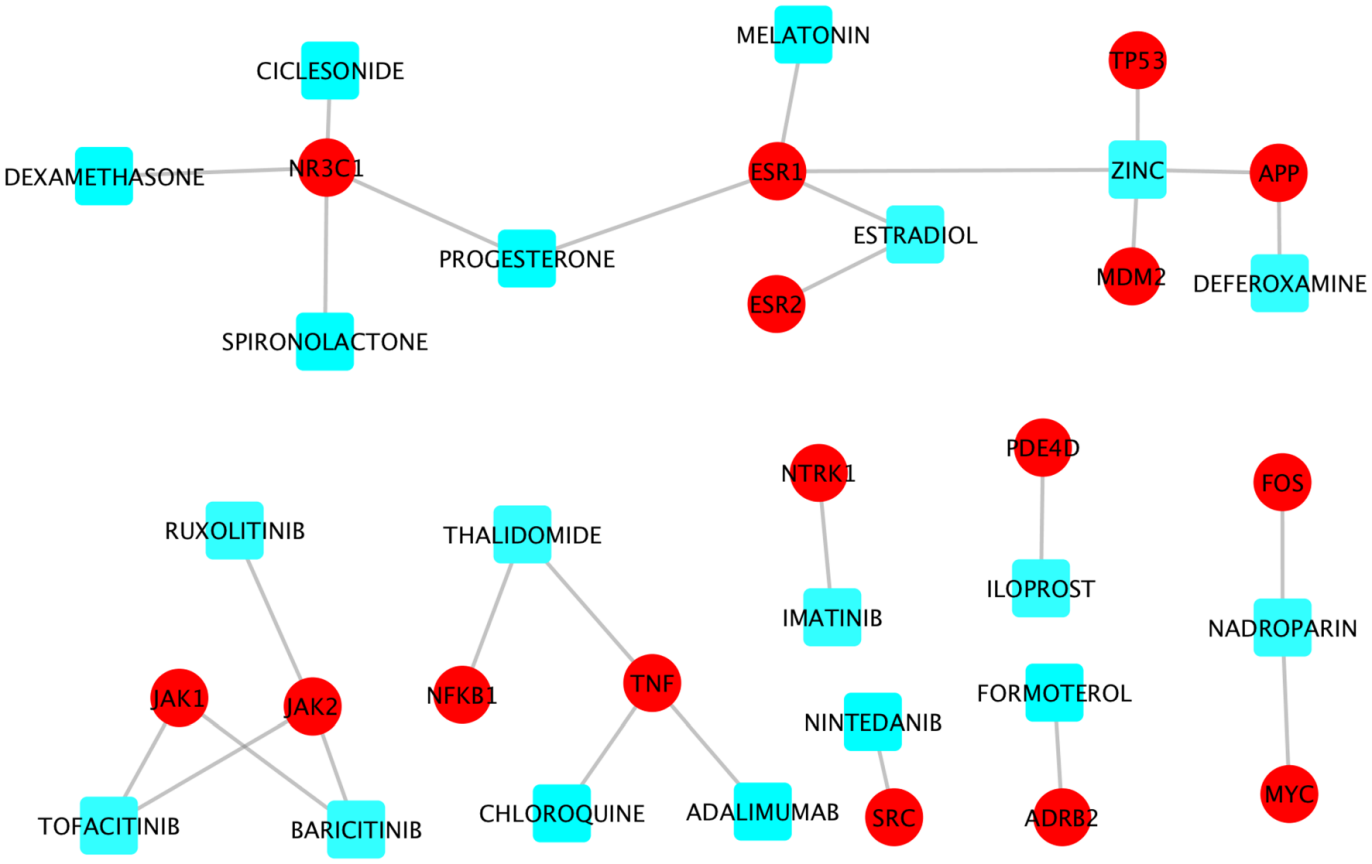
Drug-target information of 19 clinical trials drugs perturbing the RdRp-host interaction signaling network.

There were 200 genes with node degree ≥ 10 in at least one of the two RdRp-host interaction signaling networks (see supplementary Table  3). These genes can be potentially important targets that are synergistic with the RdRp for the purposes of inhibiting viral replication. To identify drugs that can inhibit the targets on this signaling network, drug-target interactions were derived from drugbank^23^ database. Among the 200 genes, there were 65 genes that had at least one drug or investigational agent inhibiting them. Of note, amongst these drugs, 19 were reported in literature concerning clinical trials of potential COVID-19 treatments (i.e., adalimumab, progesterone, spironolactone, chloroquine, imatinib, deferoxamine, estradiol, formoterol, thalidomide, melatonin, iloprost, dexamethasone, ciclesonide, zinc, nadroparin, ruxolitinib, tofacitinib, nintedanib, and baricitinib) (see Fig. 5). Interestingly, nadroparin, the MYC and FOS inhibitor, can stop the amplification of the fibrin clotting cascade. Similarly, thalidomide is a NFkB and TNF inhibitor that can be immune-suppressive and anti-angiogenic^42^. Estradiol and melatonin^43^ are ESR1 agonists that tested in clinical trials. Also, dexamethasone, NR3C1 inhibitor, was the first drug reported to be able to significantly improve the mortality rate of COVID-19 patients in clinical trials^7^. The newly clinical trial results showed that Baricitnib, the JAK2 inhibitor, was synergistic with Remdesivir to improve the outcome of COVID-19 patients^37^.

Moreover, the selected drugs (some targets have many drugs directly interacting with them) that can perturb one of the 65 signaling targets were shown in Supplementary Fig. 2. Some targets have been reported in the current literature as being potentially important and effective targets for COVID-19 treatment (in addition to drugs that could modulate the effects of cytokines), like HDAC2/3^44,45^, JAK2^46^, CDK2^47^, AKT1^48^, MDM2^49^, TNF^50^, MAPK14^17^, STAT3^51^, BCL2^52^, NFKB1A^53^, EGFR^51^. Inhibitors (drugs) of these targets can be potentially synergistic with Remdesivir to inhibit viral replication.

### Novel evidence showing that estrogens is an essential sex factor associated with the distinct fatality patterns between male and female COVID-19 patients

**Figure 6:**
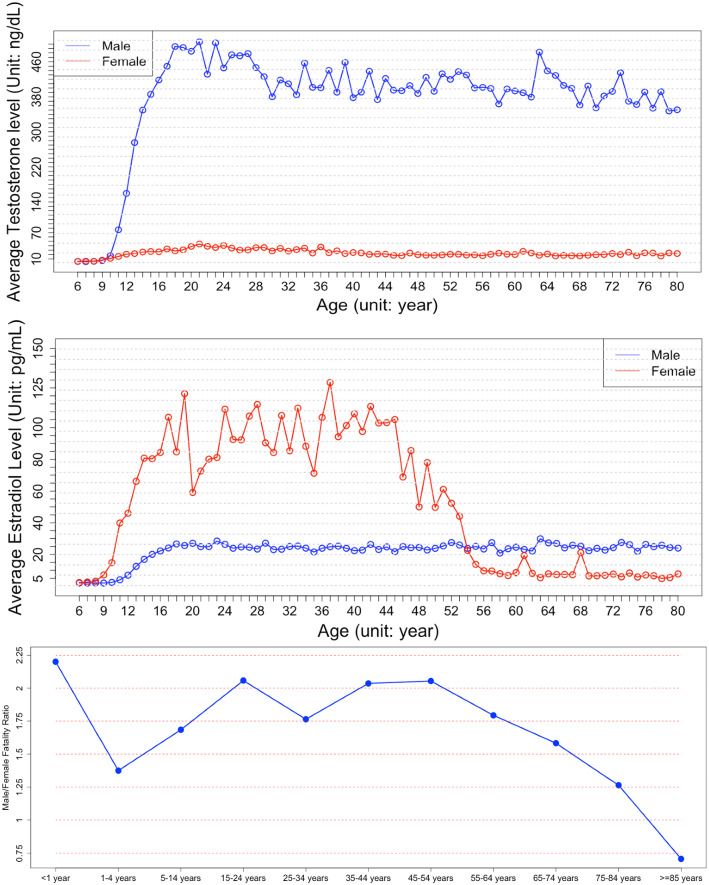
The testosterone (upper-panel) and estradiol (middle-panel) lifespan changes in women and men; and the curve of ratio between female and male mortality rate of COVID-19 patients (bottom-panel).

As shown in the results, the estrogens can inhibit the activated inflammation and immune response biomarker genes; and the ESR1 and ESR2, the signaling receptors of estrogens, were identified in the viral-host signaling network and intensively interact with other signaling targets on the network. All the results indicated that the estrogens, interacting with ESR1/ESR2, are the essential sex factors protecting young female COVID-19 patients. However, the fatality rate of old female COVID-19 patients dramatically increased. To further understand the mechanism of estrogens, we investigated the lifespan changes of the estrogens level in women and men^54^. Very importantly, it showed that the estrogens level in female reduces to an extremely low level after about 55 years of age, starting from about 45 years of age (see Fig. 6-middle panel). Of note, we further downloaded the mortality data of male and female (see Fig. 6-bottom panel) in U.S. from the CDC website^55^ (by December 31, 2020), and compared those data with the estrogens levels of female over their life span^54^ (see Fig. 6-upper panel). Surprisingly, the trends of the two curves closely approximated each-other (i.e., the curve of ratio between female and male mortality rate of COVID-19 patients vs the cure of levels of female). This specific finding might be able to explain, in terms of the molecular mechanism, the mounting evidence suggesting that men have a significantly higher mortality rate when positive for COVID-19 than young women^56^. In other words, the low level of estrogens in female before 9 years old, and the dramatic decreasing of estradiol after ~ 55 years of age is associated with an increasing COVID-19 mortality rate in female. In addition, the relative survival rate does not decrease in the 45–54 group, whereas the estradiol level starts to drop from age 45 onward in women (see Fig. 6). It indicates that the lower estrogen level can still provide survival benefits in COVID-19. Since studies reported the anti-inflammation effects of androgens^57,58^, we further analyzed the Testosterone (androgen) level change over life in female and male (see Fig. 6). Compared with the estradiol (estrogen), the decreasing speed of testosterone in male is much slower, which indicates androgens are less effective at modulating the inflammation/immune response than estrogens. In summary, these supportive data may serve an indicator that estrogens can serve as an effective treatment, especially for old male or female patients, or in combination with remdesivir and other drugs for COVID-19 treatment.

## Discussion

Millions of people are being infected by SARS-CoV-2 globally, with thousands of ensuing deaths every day. There is currently a lack of effective treatments to reduce the mortality rate of COVID-19. Remdesivir is a widely used and promising drug inhibiting the RdRp protein. However, the evidence of efficacy of using remdesivir alone is limited. From a large number of clinical trials, as aforementioned, a few exiting FDA-approved drugs, like dexamethasone and baricitinib, as their combinations, were identified to be able to reduce the death rate of patients with severe COVID-19. In addition to the RdRp target, the main protease (Mpro) target of SARS-CoV-2 was identified as another important target mediating viral replication. The new drug nirmatrelvir, inhibiting Mpro, was developed and authorized by FDA by December 2021 for the treatment of mild-to-moderate COVID-19 patients. However, the death rate for such patients remains high. Thus, the need for novel targets, and new and effective treatments, used alone or combined with these aforementioned drugs, is both critical and unlikely to cease to be a priority for the foreseeable future.

Sex had been indicated as an essential factor in the mortality of COVID-19. The global data of COVID-19 showed that men have a higher mortality rate than women. In the recent retrospective cohort studies^59,60^, estrogens supplement or replacement therapy were associated with reduced mortality in COVID-19. For example, using a nationwide cohort of Sweden^59^, estrogen supplement/treatment in postmenopausal women is associated with a decreased risk of dying from COVID-19. In another recent study^60^, it reported the protective effect of estrogen replacement therapy (hormone replacement therapy, HRT) within 6 months before the diagnosis of COVID-19. Specifically, the HRT was associated with a lower risk (odds ratio (OR) = 0.15 for unadjusted models, and OR = 0.22 for the adjusted models) of all-cause mortality in COVID-19. Therefore, the sex difference indicated that sex related genetic factors could be an interesting and novel mechanisms and targets for COVID-19 treatment, combined with existing treatment options. However, in the retrospective association studies, the underlying targets and signaling pathways associated with the estrogen/sex difference remain unclear. Compared with these association studies, we identified novel evidence and associations between estrogen and distinct mortality patterns in different age groups in female and male COVID-19 patients, by using systems biology network analysis models that integrate diverse COVID-19 related transcriptomics, proteomics, signaling pathways, drug targets, and gene ontologies (see methods). The lifespan estrogen and androgen levels of female and male were also identified and compared with the relative mortality rate of female and male COVID-19 patients. It indicated that estrogens are more effective at modulating the inflammation/immune response than androgens. In summary, estrogens have the protective effect and can prevent mortality of COVID-19 patients.

Moreover, these results can be further utilized to investigate the unknown molecular mechanism that is driving the estrogen-dependent inhibition of inflammation during COVID-19 using the uncovered signaling targets and network. Experimental validations are further needed in the context of animal models that have been ovariectomized or received additional estrogen. Though, our findings have limited clinical impact on the current pandemic, additional research including studies measuring estrogen levels and outcomes of SARS-CoV-2 infection or vaccination will help clinical practice during future outbreaks and pandemics.

There are some limitations of our analysis results. First, the current computational model is limited to separate the targets that are essential for viral replication; and the targets that are activated to fight and inhibit the infection, in the identified signaling targets/pathways associated with COVID-19. Ideally, medications or cocktails are preferred to inhibit the viral replication related targets and boost the anti-viral signaling targets. However, it is not a trivial question considering the complex interactions among these signaling targets. Thus, novel computational models are needed to identify the essential viral replication-related targets and anti-viral targets. Secondly, compared with healthy lung epithelial cells in the physiological site of infection, some targets or signaling pathways may have been altered in lung cancer cells, which might adopt some immune evasion as well as altered cell signaling. These alterations may affect which genes are upregulated as a response to SARS-CoV-2 infection (cancer cell specific). The transcriptomic data of Vero E6 cells before (3 samples) and after (24 h, 3 samples) SARS-CoV-2 infection was generated in the study^61^, and publicly available at GEO database (ID: GSE153940). We downloaded the data (raw counts), and used the DESeq2 to identify the up-regulated genes. Among the 299 identified essential genes in our study, 236 genes were measured in the Vero E6 data. And, ~ 55% (136 out of 236 genes) are also up-regulated (with p-value ≤ 0.05 and fold change ≥ 2.0); and ~ 74% (175 out of 236 genes) are up-regulated (with fold change ≥ 1.25). The comparison indicated that most of the genes might not cancer cell-specific. Whereas, some genes might be cancer cell-specific. Thus, experimental validations might be needed to further investigate these targets. Thirdly, some potential drugs with opposite mechanisms were top-ranked. It might be caused by false positive predictions. Therefore, it is important to investigate these drugs with additional drug information or evaluate these drugs using experiments. Fourth, the mechanisms of estrogens, and their relationships with other sex factors^12^, for example, the higher number of CD4+/8+ T cells, effective B cells, and higher level of type 1 interferon (IFN) should be further investigated, by combining computational and experimental models, to identify novel therapeutic targets and medications to reduce the mortality rate of COVID-19. For example, as seen in the results, after estrogens drop to very low level after 55 years of age, the female still has a lower mortality rate than male (see Fig. 6) before 85 years of age, which indicates the complex molecular mechanisms of estrogens and other sex factors.

## Conclusion

In this study, we identified that estrogens are essential sex factors associated with the distinct fatality rates between male and female COVID-19 patients. Specifically, a high level of estradiol protecting young female COVID-19 patients, and estrogens loss to an extremely low level in females after about 55 years of age is associated with the increased fatality rate of women. It is interesting and important to further investigate the molecular mechanisms of how estrogens inhibit inflammation and immune response in COVID-19. In addition, the uncovered essential host targets and treatments can be helpful to design experiments to investigate the unknown molecular mechanisms, and uncover effective targets and drugs along or combined with existing drugs as novel treatment regimens for COVID-19.

## Supplementary Information

Supplementary Figures.

Supplementary Tables.
